# *P. aeruginosa* interactions with other microbes in biofilms during co-infection

**DOI:** 10.3934/microbiol.2023032

**Published:** 2023-08-10

**Authors:** Manuela Oliveira, Eva Cunha, Luís Tavares, Isa Serrano

**Affiliations:** 1 CIISA—Center for Interdisciplinary Research in Animal Health, Faculty of Veterinary Medicine, University of Lisbon, Avenida da Universidade Técnica, 1300-477 Lisboa, Portugal; 2 Associate Laboratory for Animal and Veterinary Sciences (AL4AnimalS), 1300-477 Lisboa, Portugal

**Keywords:** antimicrobial resistance, diabetic foot wounds, cystic fibrosis, colorectal cancer, microbial interactions, otitis media, polymicrobial biofilms, *Pseudomonas aeruginosa*, spatial arrangement, *Staphylococcus aureus*

## Abstract

This review addresses the topic of biofilms, including their development and the interaction between different counterparts. There is evidence that various diseases, such as cystic fibrosis, otitis media, diabetic foot wound infections, and certain cancers, are promoted and aggravated by the presence of polymicrobial biofilms. Biofilms are composed by heterogeneous communities of microorganisms protected by a matrix of polysaccharides. The different types of interactions between microorganisms gives rise to an increased resistance to antimicrobials and to the host's defense mechanisms, with the consequent worsening of disease symptoms. Therefore, infections caused by polymicrobial biofilms affecting different human organs and systems will be discussed, as well as the role of the interactions between the gram-negative bacteria *Pseudomonas aeruginosa*, which is at the base of major polymicrobial infections, and other bacteria, fungi, and viruses in the establishment of human infections and diseases. Considering that polymicrobial biofilms are key to bacterial pathogenicity, it is fundamental to evaluate which microbes are involved in a certain disease to convey an appropriate and efficacious antimicrobial therapy.

## 1. The concept of biofilm

In nature, microorganisms can appear in either a planktonic or sessile state. In the planktonic state, bacteria move freely, whereas in the sessile state, bacteria are frequently attached in multicellular aggregates, forming biofilms [1].

The first simple model of biofilm infection was proposed by Costerton and Stewart in 1999 [2], in which polymicrobial communities of bacteria are able to produce virulence factors leading to infection. The model puts an innovative bacterial strategy into evidence and allows the understanding of chronic infections at the biochemical and cellular levels, in which antibodies [3] and white blood cells [4] are ineffective in combating biofilms. Nowadays, the models are more comprehensive; however, a broader knowledge of how polymicrobial infections by biofilms progress will allow for the elaboration of more realistic models [5].

Biofilm formation is a multistep process whereby heterogeneous communities of microorganisms are embedded into a self-produced hydrated matrix. This matrix is formed by an extracellular polymeric substance often consisting of polysaccharides, proteins, glycoproteins, and extracellular DNA, conferring the capacity of microorganisms to adhere to either biotic or abiotic surfaces [6],[7]. A vast number of human polymicrobial diseases are spread through abiotic surfaces, such as intravenous and urinary catheters, ventilator tubes, pacemakers, and orthopedic devices [8]–[10], which can be colonized by potentially pathogenic microorganisms [11]. Bacteria are the main type of microorganisms involved in biofilm formation. However, many filamentous fungi and yeasts have been associated with biofilm formation, and *Candida albicans*, a commensal mucosal organism, remains the most widely studied biofilm-producing fungus. A few viruses, like the influenza A virus, and respiratory syncytial virus (RSV), have also been found in biofilm communities [12].

Biofilms are polymicrobial communities, organized in space. Traditionally, biofilms are considered to be formed by hundreds of thousands of cells encased in a matrix and attached to a surface; however, they can also be formed by dozens of cells simply arranged in small aggregates [1].

Polymicrobial biofilms are prevalent throughout the human body, both in health and disease status, and their exoproducts can help bacteria aggregate and therefore reduce the efficacy of high-dosage antimicrobial therapy [13],[14]. Broad-spectrum antibiotics are generally used to treat polymicrobial infections, however, the recovery rate of affected individuals is low [15],[16]. Cells within the biofilm may belong to different bacterial species but are functionally equivalent pathogroups [17], developing quorum sensing systems through which they are able to communicate [18], and controlling bacterial pathogenicity and mutual growth in a specific environment [11],[19].

The interactions between microorganisms within biofilms profoundly affect disease severity and progression [20]. Although some infections require colonization by multiple interacting microorganisms, as in the case of colonization of the oral cavity by *Porphyromonas gingivalis* and commensal oral microorganisms causing periodontal disease [21], other infections are more or less severe according to the presence of specific co-infecting species, such as *Staphylococcus aureus* and *Pseudomonas aeruginosa* in chronic wound infections and cystic fibrosis (CF) [22]. The timing of polymicrobial interactions can also vary. Co-infecting pathogens can appear stepwise, with one species followed by another, or they can appear concurrently in the host [23]. Occasionally, commensal strains have a more prominent role in the disease progression than a well-known pathogen, that can participate only as a minor player, as observed for *Streptococcus mutans* in dental caries [24].

## 2. Interactions and spatial arrangement in polymicrobial biofilms

Bacteria within polymicrobial biofilms can benefit from each other and facilitate cohabitation on epithelial surfaces through the efficient use of metabolic by-products, and the production and secretion of beneficial molecules [25]. Beyond cooperating, they can also compete with each other for resources and space by producing toxins or other extracellular metabolites [26]. The mechanisms that allow these bacteria to interact *in vivo*, including cooperative and antagonistic behaviors between biofilm-specific communities, are not fully understood, but are known to be strongly influenced by the host and its immune system, and by chemical and physical interactions, including cell-cell communication via the bacteria's quorum-sensing cross talk [27]. The events associated with the establishment of these relationships include contact-dependent attachments, an increase in colonization, immunomodulation, and expression of virulence potential [11],[28].

Polymicrobial infections can be synergistic if the interactions between microbial strains or species result in an outcome that is greater than the sum of the one from individual microorganisms. Therefore, synergic infections are associated with more severe consequences for the host [29].

An example of a polymicrobial synergistic environment is the oral cavity, since oral microbes generally only exist there, and so the interactions between them have positively evolved [30]. On the other hand, wounds are polymicrobial synergistic environments where microbes, such as *P. aeruginosa* and *S. aureus*, have evolved in different habitats interacting over time with other microbes. *P. aeruginosa* main habitats are soil and water, while for *S. aureus* is the respiratory tract and the skin of humans and animals [22]. However, over time, *P. aeruginosa* started to colonize the same habitats as *S. aureus* [31]. The interaction between these bacterial species is sometimes cooperative but mostly antagonistic, and the outcome of their interactions is synergistic, leading to chronic infections resistant to antimicrobial therapy [32].

It is known that microorganisms are not accidentally distributed throughout the body; for example, the endemic *Helicobacter pylori* only resides in the stomach. Additionally, the same body sites of different individuals are usually more similar in microbial composition than those from different sites of the same individual [33]. These examples are studied by biogeography, which uncovers the distribution of species through space and time, including the spatial organization of microbial communities in biofilms, that depends on different ecological and evolutionary forces [23],[34],[35].

**Figure 1.**
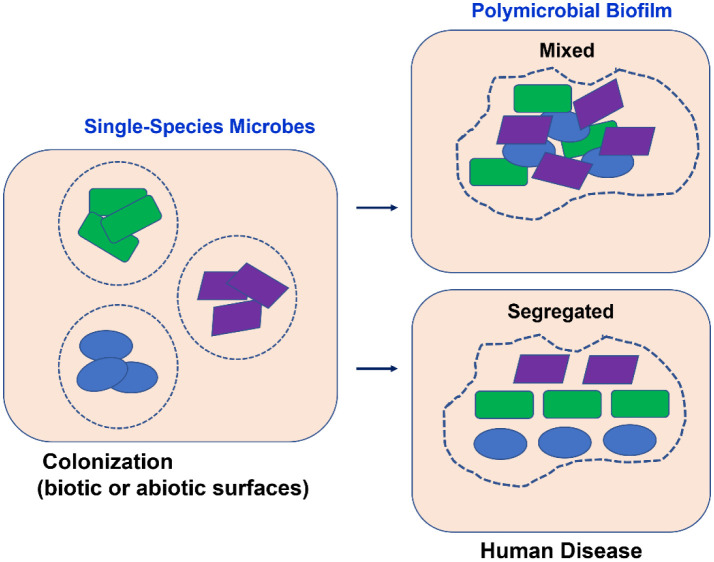
Single species microbes colonizing the host cells and the formation of segregated or mixed polymicrobial biofilms underlying disease development.

Microbial community organization can be classified as either spatially mixed or segregated, with the former being associated with enhanced interactions between microbes and the latter with attenuated interactions. Both types of spatial organization and their associated polymicrobial relations can influence virulence and infection, and according to Stacy *et al*. [23], they are divided into four categories: two types of cooperative interactions, 1. physical with a mixed organization, and 2. chemical with a mixed organization; and two types of antagonist interactions, 3. chemical with a segregated interaction, and 4. physical with a segregated interaction (Figure 1).

One example of physical interaction resulting in a mixed organization is co-aggregation [23]. Co-aggregation is prevalent in the oral cavity, where intercellular binding between distinct bacterial taxa occurs. Most oral bacterial species need at least one partner to co-aggregate with in order to multiply [36], allowing for the formation of human dental plaque [37]. For example, *Veillonella atypica* can only grow in saliva if co-aggregated with streptococci [38], and *Streptococcus gordonii* can only multiply in low-arginine media if co-aggregated with *Actinomyces naeslundii* [39]. Most co-aggregation cases are mutualistic, though there are also examples of parasitic interactions [40].

Local growth promotion is an example of a chemical interaction resulting in a mixed organization [23]. Chemical interactions generally occur when bacteria are located near each other and when the protector pathogen is at a high density. The level of mixing in biofilms formed by strongly interdependent species is higher than in those formed by weakly interdependent species, and even more so than in biofilms formed by competing species [41],[42].

These interactions can promote an observed antimicrobial cross-protection, for example, when *S. aureus* is closely surrounded by a highly dense layer of ampicillin-resistant *P. aeruginosa*, shielding *S. aureus* from ampicillin action [43]. In addition, these biofilms have the capability to facilitate metabolic cross-feeding. For instance, *Aggregatibacter actinomycetemcomitans* utilizes the lactate produced by *S. gordonii*. This relationship is synergistic because, in turn, *A. actinomycetemcomitans* clears peroxide, a by-product of *S. gordonii* metabolism, maintaining a gap larger than 4 µm from *S. gordonii* to avoid the growth inhibition by peroxide, resulting in higher microbial burdens in the presence of oral abscesses and periodontitis [23],[44]. Other synergistic oral pathogens also show identical patterns [45],[46].

Local growth inhibition by a chemical antagonist interaction causes spatial segregation [23]. Chemical signals act at a very short-range (smaller than 10 µm), generating fine-scale spatial organization. Generally, signals with no need for a specific receptor are more widespread. For example, many streptococci produce lactate and hydrogen peroxide as waste products that concentrate in the surrounding area. Hydrogen peroxide can reach concentrations in the millimolar range at a distance of 100 µm from *S. gordonii* biofilm [47]. These products act as broad-spectrum toxins, eliminating local competitors and consequently regulating fine-scale segregation patterns.

Another example is the production of pyocyanin by *P. aeruginosa*. In chronic wounds, this virulence factor senses cell wall fragments discarded by *S. aureus* [22] and contributes to the elimination of this bacterial species when located in the proximity. The spatial organization of these bacteria is highly segregated, with *S. aureus* generally present only at a distance of 20–30 µm from the wound surface, and *P. aeruginosa* in deeper depths, at around 55 µm from the wound surface [48]. Because the wound environment is highly viscous and restrictive for cell migration, it avoids species mixing and further killing, allowing *S. aureus* and *P. aeruginosa* to co-infect wounds [26].

Biofilm remodeling is an example of physical interactions resulting in a segregated organization [23]. This is due to either the production or breakdown of extracellular matrix components, which contributes to the segregation of microbial members. For example, when growing on an agar surface, *Pseudomonas fluorescens* regularly produces mucoid variants that overproduce exopolysaccharides. Interestingly, these variants can move to the top of the biofilm, gaining access to oxygen and restraining their competitors [49]. In CF patients, *P. aeruginosa* also produces mucoid variants that are able to overproduce the polysaccharide alginate, interfering with the presence of *S. aureus* and *Burkholderia cenocepacia* [50],[51], creating a highly marked organization [52]. Other major *P. aeruginosa* polysaccharides, such as Pel and Psl, also have different roles in the spatial organization of biofilms [53]. The re-positioning of polymicrobial members in biofilms can influence community interactions and disease progression [23].

It has been observed that cells within a biofilm can detach and disseminate into the surrounding environment [12],[54]. It was suggested that biofilm dispersion is an effective mechanism for disseminating bacteria with reinforced colonization properties and pathogenic potential in the host environment. The factors that promote biofilm formation and dispersal have been studied mostly in Gram-negative bacteria and involve the degradation of matrix components, such as polysaccharides, proteins, and extracellular DNA, the occurrence of local cell lysis, and the production of surfactant molecules that reduce surface tension. The dispersion of biofilm cells is essential to maintaining the infection, promoting its recidivism and chronicity [12],[54].

Interestingly, biofilm assembly can be driven by two processes that are independent of the bacterial genes present, and intrinsic to the physical properties of the host-derived macromolecules such as mucus: depletion aggregation and bridging aggregation. In both mechanisms, the bacterial functions usually required for biofilm formation, including surface adherence, synthesis of the extracellular matrix, cell-to-cell communication, and motility, are not involved [126]–[128]. In lung infections, biofilm assembly by *P. aeruginosa* colonizing the mucus is driven by mucin, which is abundant in chronic infections, through depletion aggregation. This non-adsorbing polymer creates mutual attraction forces between neighboring cells, creating an unbalanced osmotic pressure that pushes them together into forming ordered aggregates, which lead bacteria to become less susceptible to antibiotics [126],[127]. The higher the cell number and polymer concentration are, the greater the aggregation is [126],[127]. However, aggregation can also occur at low cell numbers when two or more non-adsorbing polymers are mixed [126],[128], as observed in the early development of CF, in which mixtures of DNA, F-actin, and mucin found in CF secretions can aggregate *P. aeruginosa* at low concentrations (10^5^ CFU/mL) [126],[128]. These studies suggest that the physical mechanisms associated with mucus contribute to biofilm assembly, and that further studies aiming to target depletion-mediated antibiotic tolerance for the treatment of chronic infections, such as CF, would be most valuable [128].

## 3. Polymicrobial infections in humans

There is a substantial diversity and concentration of microorganisms in the limited space of the human body, with evidence of their co-evolution over thousands of years. For a disease to occur, the microbial populations colonizing the host cells generate a state of dysbiosis that shifts the microbial composition from healthy to unhealthy [11],[55]. For example, *P. gingivalis* is a secondary colonizer of the gingival plaque, which harbors more than 500 bacterial species that colonize the oral cavity either permanently or transiently, producing a set of virulence factors that will benefit this species and other oral microorganisms; it is a major etiologic agent driving dysbiosis and contributing to chronic periodontitis [56]. Furthermore, bacteria forming biofilms are present in higher concentrations in disease cases. For example, biofilms are at least one hundred-fold more concentrated in biopsies from patients with inflammatory bowel disease compared with those from healthy individuals [57]. Therefore, in diseases caused by polymicrobial biofilms, the composition and concentration of the microbial community can predict the disease severity and outcome [11].

For the establishment of a disease, the inhibition of DNA repair mechanisms must occur [58],[59]; alternatively, the damage of the host cells and their DNA can occur, either indirectly through oxidative stress induced by the activation of the innate host response [60], or directly via the presence of microbial virulence factors, such as genotoxins produced by several enteric pathogens that have a strong immunomodulatory effect on the intestinal mucosa [61]–[63]. Chronic induction of severe DNA damage can lead to the activation of cellular senescence [64], by which host cells enter a permanent cell cycle arrest but maintain a high metabolic state, named the senescence-associated secretory phenotype. In this state, they are able to secrete a huge number of inflammatory cytokines, immune modulators, growth factors, and proteases [63],[65]. Cellular senescence can increase host susceptibility to genotoxin-producing organisms, such as typhoid and non-typhoidal *Salmonella*, *Escherichia coli* phylogroup B2, *Shigella* spp., and *Campylobacter* spp. [61],[62], as well as to the action of other microorganisms like *Fusobacterium nucleatum*, *Streptococcus pneumoniae*, influenza virus, and varicella zoster virus [66]–[68].

Oral cavity diseases, otitis media, keratitis, diabetic foot wound infections, chronic infection in the CF lung, pneumonia, burn wounds, osteomyelitis, urinary tract infections, vaginitis, medical device-related infections, percutaneous endoscopic gastrostomy tube infections, peritonitis [11], and certain types of cancer [63] are examples of human diseases caused by polymicrobial biofilm communities (Table 1).

**Table 1.** Diseases commonly associated with polymicrobial biofilm formation on human mucosal tissues or epidermal layers.

Human body location	Human infection or disease	Most prevalent microbial biofilm populations
Middle ear	Otitis media	*Haemophilus influenzae* (non-typeable), *Moraxella catarrhalis*, *S. pneumoniae*, and upper respiratory viruses [69]
Oral cavity	Dental Caries	*Actinomyces gerencseriae*, *Atopobium*, *Bifidobacterium*, *Enterococcus faecalis, Lactobacillus fermentum, Propionibacterium, Pseudoramibacter, Streptococcus constellatus*, *S. mutans*, *Streptococcus parasanguinis, Streptococcus salivarius*, *Veillonella* [70],[71], *C. albicans* and streptococci [72]
	Denture stomatitis	*C. albicans*, *S. aureus*, and *S. mutans* [73]
	Periodontitis	Early-onset periodontitis localized and aggressive [74]: *A. actinomycetemcomitans*, *Capnocytophaga sputigena, P. gingivalis*, *Prevotella intermedia* [75], *S. gordonii* [44], and Gram-negative anaerobic bacterium *F. nucleatum* [76] Chronic adult periodontitis [74]: *P. gingivalis*, *Tannerella forsythia*, and *Treponema denticola* [75]
Veins (e.g., superior vena cava, subclavian vein)	Parenteral nutrition feeding tube infection	Enterobacteriaceae, lactobacilli, streptococci, staphylococci, and *Candida* spp. [77]
Lung	Cystic fibrosis	*P. aeruginosa* forming dual-biofilms with *Burkholderia cepacia* [78], *S. aureus* [79], *Streptococcus milleri* group (SMG) pathogens [80], *Aspergillus fumigatus* and *C. albicans* [81],[82], and RSV virus [83] Also present are: *H. influenzae, Stenotrophomonas maltophilia* [84], *Actinomyces, Prevotella*, *Propionibacterium*, and *Veillonella* [85]
Colon	Familial adenomatous polyposis	Proteobacteria and Bacteroidetes, specifically *E. coli* and *Bacteroides fragilis* [86],[87] .
	Colorectal cancer	*B. fragilis* and oral pathogens including *F. nucleatum*, *Parvimonas micra*, and *Peptostreptococcus stomatis* [86],[87]
Urinary tract	Urinary tract infections	*Klebsiella pneumoniae* and *Proteus mirabilis* [88], also *Acinetobacter baumannii, E. coli*, *S. aureus*, *Staphylococcus epidermidis, P. aeruginosa* [89]
Foot wounds	Diabetic foot infections	*Corynebacterium* spp., *Enterococcus*, *E. coli*, *S*. *aureus*, *S. epidermidis, Porphorymonas spp., Prevotella spp*., *Pseudomonas* [90], *Bacteroides* spp., *Clostridium* spp., and *Fusobacterium* spp. [91] It is important to refer that, several species are unidentifiable by standard culturing techniques [90]

In the next chapter, some examples of polymicrobial biofilm-mediated infections occurring in different regions of the human body will be discussed, mainly focusing on biofilm formation, including otitis media, colorectal cancer, diabetic foot wound infections and CF.

## 4. Examples of polymicrobial biofilm-mediated infections and diseases

### 4.1. Otitis media and related nasopharyngeal infections

Nasopharyngeal infections are related to otitis media. The eustachian tube connects the nasopharynx with the middle ear cavity; as such, an infection in the nasopharynx may spread through this tube to the middle ear, prompting otitis media [92]. Frequently, most cases of otitis media are spontaneously resolved within a few weeks, but chronic infections can lead to partial or total hearing loss [93].

The microbial community is established in the nasopharynx in the first year of life, varying during individual's lifetime [94]. The microbial species responsible for otitis media are the commensal bacteria normally found colonizing this region, such as *S. pneumoniae*, non-typeable *H. influenzae*, and *Moraxella catarrhalis*, along with upper respiratory viruses, including influenza A virus, RSV, human rhinovirus, and adenoviruses (Table 1) [69]. Pre-infection with upper respiratory tract viruses predisposes the host to disease development and enhances the otitis media progress [95] by changing the physiological properties of infected airways and modulating the immune response [11],[96]. Bacteria shift from colonizers to invaders causing infection, often as polymicrobial biofilms, when there is either an epithelial rupture or immune dysfunction [97].

Concerning biofilm formation by *S. pneumoniae*, this species starts by attaching to the substratum through the mediation of pneumococcal adhesion factors, such as PspA, CbpA, and PcpA [98], followed by pneumococcal aggregation and matrix formation and maturation [99],[100]. Events like cellular autolysis, genetic competence, and biofilm formation are regulated by two quorum sensing systems, Com and LuxS/AI-2, in an intercrossed way [101], through the secretion of strain-specific competence-stimulating peptides, which allow for the release and uptake of DNA from other streptococci present in the biofilm [102]. Competence is highly efficient for spreading β-lactam resistance determinants between the streptococcal species [100],[102]. In polymicrobial biofilms combining *S. pneumoniae* and *H. influenzae*, it has been found that susceptible *S. pneumoniae* strains can gain protection against antibiotics through the passive transference of β-lactamase genes from resistant *H. influenzae* [103]. A few other streptococcal species that colonize the pharynx, such as *S. intermedius*, *S. oralis*, *S. gordonii*, and *S. mutans*, use LuxS/AI-2 to regulate competition or cooperation mechanisms among species present in the pharynx and to control biofilms [104].

### 4.2. Colorectal cancer

Polymicrobial biofilms have been described in proximal colorectal cancer and in polyps of patients with familial adenomatous polyposis, a hereditary condition caused by mutations in the tumor suppressor gene, adenomatous polyposis coli [86]. Of note, patients with biofilm tumors in the colon possess analogous structures in distal regions that are tumor-free, though they present significant pro-tumorigenic changes on the underlying colonic epithelium [105]. As such, it can be inferred that the alteration of the colon microbiota precedes tumor formation, and that colon mucosal biofilm detection may predict an increased risk for the development of colorectal cancer and adenomas [63],[86].

Biofilms in familial adenomatous polyposis are mainly formed by *E. coli*, *Bacteroides fragilis*, and other *Proteobacteria* and *Bacteroidetes*, whereas biofilms in colorectal cancer are composed of *B. fragilis* and oral pathogens, including *F. nucleatum*, *Peptostreptococcus stomatis*, and *Parvimonas micra* (Table 1) [86],[87].

In familial adenomatous polyposis, there is a synergistic effect when *E. coli* and *B. fragilis* biofilms co-colonize the colon, which is associated with an enhanced morbidity and mortality [63],[86]. In this case, the carcinogenic properties are related to two key virulence factors. The first is a zinc-dependent metalloprotease toxin with a mucolytic effect secreted by some enterotoxigenic *B. fragilis*, which promotes a colonic inflammatory condition [106], creating favorable conditions for the formation of specific polymicrobial biofilms. The second is the expression of a functional *psk* island of *E. coli*, which encodes the genotoxin colibactin, which promotes DNA damage and contributes to disease progression [59].

### 4.3. Diabetic foot wound infections

Diabetes mellitus is characterized by a defective physical response to insulin. It affects nearly 6.4% of the worldwide population, and since this number is estimated to double by the year 2030, the Centers for Disease Control and Prevention classified diabetes as a current epidemic [107]. People with diabetes mellitus have a 15–25% estimated probability of developing a diabetic foot ulcer (DFU) [108], and among DFU patients, 80% will suffer lower-limb amputations due to an ulcer infection [109]. Generally, DFU are infected with polymicrobial biofilms [17],[110],[111], including opportunistic pathogens [111] and anaerobic bacteria [112],[113], that are associated with delayed healing of these chronic wounds [114] and an increased risk of death within 18 months [115]. There are several studies based on different methods, from conventional culture data to genomic approaches, aiming at establishing the microbe population present in infected DFU. According to Citron *et al*. [91], among positive cultures, approximately 16% contain one bacterial species, 20% contain two species, 20% contain three species, 13% contain four species, and 30% present five or more bacterial species. Additionally, among the microbes identified, 49% are anaerobes, with *Fusobacterium* spp., *Porphyromonas* spp., *Prevotella* spp., *Bacteroides* spp., and *Clostridium* spp. being the most frequently detected. Most anaerobic species can be found alongside aerobic organisms [91],[116], suggesting that the former may play a significant role in the etiology of chronic wound infections, helping to control bacterial pathogenicity and biofilm formation as functionally equivalent pathogroups [1],[17],[112]. The most predominant aerobic and facultative anaerobic species identified in different studies include *Pseudomonas* spp. (16%), *E. coli* (14.6%), methicillin-susceptible *S. aureus* (13.3%), and *Streptococcus pyogenes* (10.6%) [112],[117].

In DFU, the hypoxic environment deeply influences bacterial diversity and localization, being observed at a higher occurrence of strictly anaerobic bacteria and some Proteobacteria in deeper ulcers [48],[118], making each DFU a unique microbial complex [119]. DFU duration is directly correlated with the bacterial diversity present in the wounds and with the Proteobacteria frequency, and is indirectly correlated with the frequency of staphylococci [120]. Additionally, the presence of fungi in polymicrobial biofilms is associated with a poor diagnosis and delayed healing of chronic wounds [121].

In DFU, some polymicrobial communities have a higher ability to produce biofilms than others. According to Mottola *et al*. [122] biofilms formed by *P. aeruginosa* plus *Enterococcus* spp., *Acinetobacter* spp. plus *Staphylococcus* spp., and *Corynebacterium* spp. plus *Staphylococcus* spp., have a higher biofilm-producing ability than those formed by *E. faecalis* plus *Staphylococcus* spp. and *E. faecalis* plus *Corynebacterium* spp. The authors have also demonstrated that these polymicrobial biofilms are able to synergistically produce higher biofilm concentrations and virulent factors than individual species [122].

### 4.4. Cystic fibrosis of the lung

CF is an autosomal recessive genetic disorder that is most common among Caucasians. The majority of CF cases are due to an inherited mutation in phenylalanine residue 508 of a specific chloride ion channel, named the cystic fibrosis transmembrane conductance regulator (CFTR) [123]. However, there are approximately 1,900 known mutations associated with CF (http://genet.sickkids.on.ca/app). In CF, there is a Na^+^ and Cl^−^ ion imbalance that causes water to be retained inside the cells, leading to dehydration of the extracellular space [124]. This creates a viscous and thick mucus layer that cannot be easily cleared by cilia and that traps bacteria, resulting in extensive mucous accumulation and severe lung infection [125]. Lung infection caused by polymicrobial biofilms remains the primary cause of morbidity and mortality in CF patients [84].

*H. influenzae* and *S. aureus* are initial colonizers of the lungs of young children with CF [129],[130]; by the age of twenty, 60–70% of CF patients present intermittent and chronic colonization by *P. aeruginosa* [131]; and, eventually, the lung is terminally colonized by *B. cepacia* (Table 1) [132]. *P. aeruginosa* and *B. cepacia* rarely infect healthy human lungs [133], making them later colonizers. The consensus is that *P. aeruginosa* surpasses *S. aureus* in the resident microbial community at a later age and becomes the prevalent pathogen in the CF lung [123],[134],[135]. In fact, it is the dominant microorganism in at least 50% of adult CF patients [136]. However, recent studies demonstrated that *S. aureus* is often co-isolated with *P. aeruginosa* in at least 30% of adult CF patients [137]–[139].

CF infection with *P. aeruginosa* has been associated with more rapid lung failure, the worst clinical results, and premature death [135],[139],[140]. Moreover, CF patients infected with *P. aeruginosa* are more vulnerable to developing secondary infections, for example with species from the *Burkholderia cepacia* complex, which are associated with cepacia syndrome. This syndrome predisposes patients to usually fatal necrotizing pneumonia [51],[141].

The *B. cepacia* complex consists of several phenotypically indistinguishable genomovars (i.e., different species that are phylogenetically closely related) [142]. Its members form mucoid biofilms, engaging in a close network of interactions with *P. aeruginosa*, which promote the exchange of genetic material [50], prompting a more rapid decline in pulmonary function [78].

Additionally, fungi and yeasts inhabit the individuals' airways, in which *Aspergillus fumigatus* and *C. albicans* are the most prevalent fungi and yeast, respectively [82], being identified in up to 50% of CF patients (Table 1) [143],[144]. These fungi are able to form biofilms with *P. aeruginosa* [31],[135],[145],[146]. Moreover, the metagenomic characterization of the lung microbiome allowed for the description of new pathogens in the CF lung, such as *Ralstonia mannitolilytica*, which is associated with accelerated disease progression and increased mortality [147]. More than 450 viral genotypes were also described [148], some of them being related to the onset of CF pulmonary exacerbations [149]. Interestingly, CF biofilms consisting of *P. aeruginosa* and either the anaerobic emerging species *Inquilnus limosus* or *Dolosigranulum pigrum*, rarely observed elsewhere, are found to present an increased resistance to most antibiotics [150].

The role of multispecies interactions is of paramount importance in predicting patients' health. In the next chapter, the role of biofilm-species interactions in shaping virulence, antimicrobial resistance, and disease progression will be discussed.

## 5. Interaction between pairs of pathogens in biofilms during co-infection

*P. aeruginosa* is extremely difficult to eliminate mainly due to the low permeability of its cells, attributed to the gram-negative cell wall, added to the presence of efficient efflux systems from five superfamilies (major facilitator superfamily, ATP-binding cassette superfamily, resistance nodulation division family, small multidrug resistance family, and multidrug and toxic compound extrusion family), the expression of intrinsic and acquired antibiotic resistance mechanisms, the ability to express a considerable number of extracellular virulence factors, and the ability to adapt to several environmental conditions [151].

The ESKAPE group is the acronym for *Enterococcus faecium*, *S. aureus*, *K. pneumoniae*, *A. baumannii*, *P. aeruginosa*, and *Enterobacter* spp., which are highly virulent and antibiotic resistant pathogens [152]. When *P. aeruginosa* forms biofilm complexes with microorganisms belonging to the ESKAPE group, such as either *S. aureus* or *A. baumannii*, its elimination is almost unachievable, leading to treatment failure in diseases like CF, urinary tract infections, or chronic wound infections (Table 1) [153]. Therefore, the mechanisms behind the interaction between this bacterial species and other microbes' have been the subject of many studies. Next, the interaction between *P. aeruginosa* and other microbes in biofilms during co-infection will be described.

### 5.1. P. aeruginosa and S. aureus

*P. aeruginosa* and *S. aureus* are highly prevalent pathogens found in chronic wound infections [26], including in diabetic foot infections [25],[122], surgical site infections, necrotizing fasciitis [154],[155], burn wounds [156], and CF [157].

Studies regarding wound infections have shown that, *S. aureus* is usually located surrounding the surface of the wound and *P. aeruginosa* in the deeper sections of the wound [48],[158]. However, the location of *P. aeruginosa* in deeper regions of the wound, which may seem contradictory since it is a strict aerobic species, requires further explanation. Interestingly, according to Trizna *et al*. [159], after vancomycin treatment, *S. aureus* can be found in the middle-lower layers, i.e., biofilms' spatial organization shifts from segregated to mixed after antibiotic-induced stress. Therefore, the localization of both *P. aeruginosa* and *S. aureus* may not be segregated, being influenced by stress, which may benefit both species [159].

Although not being fully elucidated, during co-existence in polymicrobial infections, *S. aureus* and *P. aeruginosa* can potentially have both antagonistic and cooperative roles, mediated by several virulence factors (Table 2).

**Table 2.** Antagonistic and cooperative relationships between *P. aeruginosa* and *S. aureus* mediated by specific virulence factors, and their consequences.

Virulence factors		Consequences
• Antagonism/competition between *P. aeruginosa* and *S. aureus*		
Staphylolysin (LasA)		Induces lysis [160]–[162]
Rhamnolipids		
		Inhibit biofilm formation and disrupt established biofilms [162],[163]
Cis-2-decenoic acid		Inhibits biofilm formation, disrupts established biofilms, enhances metabolic activity, and reverses persistence [162],[164]
Long-chain N-acyl homoserine lactones		Inhibit bacterial growth [160],[165]
Respiratory toxins	Hydrogen cyanide	Blocks the respiratory chain [160],[166]
	Pyocyanin	Blocks the respiratory chain and generates reactive oxygen species [167]
	Quinoline N-oxides	Block the respiratory chain [168], inhibit growth, and select for small colony variants (SCV) [160],[169]

• *S. aureus* evasion from *P. aeruginosa* killing		
Formation of small colony variants (SCV)		Respiration-defective small colony variant phenotype [157],[170]
Cell wall deficient L-form-like colonies		Evade LasA mediated lysis by *P. aeruginosa* [160],[171]

• Cooperation between *P. aeruginosa* and *S. aureus*		
Exoproducts of *S. aureus*		Restore and enhance swimming and swarming motility of *P. aeruginosa* [172]
The secreted Protein A (SpA) of *S. aureus*		SpA protects *S. aureus* and *P. aeruginosa* from phagocytosis by neutrophils [173]
Mucoid phenotype by *P. aeruginosa*		Protects *S. aureus* from *P. aeruginosa* killing [174],[175], and also from antibiotic action [176],[177]

There is evidence that *P. aeruginosa* competes with *S. aureus* during early co-infections; however, in chronic long-term infections, *P. aeruginosa* may co-exist with *S. aureus*, with the presence of both bacteria reaching an equilibrium. This finding is in accordance with a previous study [178], that provides indications that chronicity increases the virulence of *P. aeruginosa*, by promoting its co-existence with *S. aureus* [25]. Importantly, *S. aureus* does not have any effect on *P. aeruginosa's* transcriptome, which suggests that this bacterial species is the main pathogen controlling infection progression [179].

During early co-infections, *P. aeruginosa* has an antagonistic behavior towards *S. aureus*, associated with the production of toxins such as staphylolysin (LasA), rhamnolipids, cis-2-decenoic acid, and long-chain N-acyl homoserine lactones [160] (Table 2). LasA cleaves the glycyl-alanine and glycyl-glycine bonds in the peptidoglycan layer of *S. aureus*, inducing bacterial cell lysis [160]–[162].

Rhamnolipids and cis-2-decenoic acid have anti-adhesive properties and promote biofilm dispersion, and together, they disrupt established biofilms and inhibit their formation by different bacteria and fungi in a dose-dependent manner [162],[164]. Moreover, cis-2-decenoic acid has been found to enhance bacterial metabolic activity and reverse the persistence state of *S. aureus*. Persister cells are phenotypic variants of regular cells representing a subpopulation of non-mutants, described as tolerant cells that can be present in both planktonic cultures and biofilms [180],[181]. Persisters are dormant metabolically quiescent cells with reduced metabolism, which enables them to tolerate high concentrations of antimicrobials [182]. Therefore, the presence of persister cells in chronic infections hampers their treatment [182],[183]. The reversal of a persister state to an antimicrobial-susceptible state can be due to a higher membrane permeability induced by cis-2-decenoic acid and the consequent increase in antimicrobial uptake [162],[164]. Regarding N-acyl homoserine lactones, they inhibit the growth of *S. aureus* in a dose-dependent manner [165]. At sub-inhibitory concentrations, they do the opposite, leading to robust biofilm formation and host cell invasion by *S. aureus* [165],[184].

*P. aeruginosa* produces several respiratory toxins, including hydrogen cyanide, pyocyanin, and mainly quinoline N-oxides, which interfere with the electron transport chain (cytochrome system), blocking the anaerobic respiration of *S. aureus* [160]. Pyocyanin is a potent phenazine, a prominent virulence factor, and a pseudomonal iron-scavenger, that induces the generation of reactive oxygen species, such as hydrogen peroxide and superoxide radicals, that can lead to cell death [167]. Quinoline N-oxides are quorum sensing-regulated virulence factors that block the oxidation of cytochrome b1 and the reduction of cytochrome a2 in *S. aureus* [168]. At higher concentrations, quinoline N-oxides inhibit bacterial growth; however, at lower concentrations, they may induce the selection of *S. aureus* SCV [160],[169].

Despite the antagonistic behavior of *P. aeruginosa*, *S. aureus* can counter-adapt to resist killing by this bacterial species by forming SCV and L-form colonies (Table 2). *S. aureus* evades *P. aeruginosa* quinoline N-oxides respiratory attack by forming SCV, which is a respiratory-defective phenotype that arises from mutations in metabolic genes [185], which is able to shift to fermentative metabolism due to insufficient oxygen availability in the biofilm [157],[170],[186]. *S. aureus* SCV are a slow-growing subpopulation of small and non-pigmented colonies, non-hemolytic, with reduced expression of virulence factors, and usually auxotrophic, i.e., they may lost the ability to synthesize certain substances, including thymidine, hemin, or menadione [160],[187],[188]. The enhanced survival of SCV is associated with a reduced activity of the accessory gene regulator (*agr*), which is a global regulator, related to the regulation of quorum-sensing systems and several virulence genes [187]. In the absence of environmental pressure, the temporary SCV phenotype can return to the wild-type phenotype [188]. To resist reactive oxygen species produced by *P. aeruginosa*, *S. aureus* SCV produce important antioxidant components, such as superoxide dismutases, catalase, and the pigment staphyloxanthin [189]. An SCV phenotype is associated with disease progression [190], as it exhibits reduced membrane potential, which is related to an increased tolerance to cationic antibiotics [135],[190], efficient evasion of the host immune system, and enhanced cell persistence [170],[187]. Therefore, the antagonistic interactions between *P. aeruginosa* and *S. aureus* mediated by quinoline N-oxides have the potential to directly influence disease progression [135],[160],[170].

To evade LasA-mediated lysis by *P. aeruginosa*, *S. aureus* may produce cell wall deficient L-form-like colonies that have lower surface charge and stronger hemolytic activity than the walled forms [171],[191]. Additionally, the aggregated characteristic of L-forms is identical to the one observed in biofilms, which could lead to enhanced persistence in the presence of stress and antibiotics [191], and promote an increased virulent infection [192].

During long-term chronic infections, *S. aureus* cooperates with *P. aeruginosa* by secreting exoproducts such as staphylococcal protein A (SpA) [160] (Table 2). Some exoproducts secreted by *S. aureus* enable *P. aeruginosa* to restore and enhance its swimming and swarming motility [172]. *S. aureus* SpA interacts with the polysaccharide locus (Psl) and the protein component of type IV pili (PilA) on the *P. aeruginosa* cell surface. When SpA attaches to Psl, this contact protects *P. aeruginosa* from phagocytosis by the host neutrophils [173]. Moreover, *S. aureus* SpA binds to the immunoglobulin Fcγ domain, preventing its own opsonophagocytosis and death. However, in the absence of Psl on the *P. aeruginosa* cell surface, SpA binds to PilA, inhibiting biofilm formation [173]. This is interesting since the genetic background of each *P. aeruginosa* strain can influence the progression of the disease, depending on either the presence or absence of Psl.

Regarding CF, it is recognized that a hallmark of chronic pulmonary infections is the presence of *P. aeruginosa* strains that are able to shift to a mucoid phenotype, due to the overproduction of the exopolysaccharide alginate, mediated by a mutation within the *mucA* anti-sigma factor [193]. Even though it is unexpected, the overproduction of alginate by mucoid strains inhibits *P. aeruginosa's* anti-staphylococcal activity, such as the production of rhamnolipids, and quinolone signals [174],[175]. Therefore, in the presence of mucoid variants, *S. aureus* strains are spared from *P. aeruginosa* killing [174], and both planktonic and sessile *S. aureus* forms are protected from antibiotic action [176],[177]. This indicates that *P. aeruginosa* shifts from a competitive to a cooperative approach during chronic CF, which benefits both species [177].

Although *S. aureus* has mechanisms to escape the toxicity and metabolic stress associated with the presence of *P. aeruginosa* and even sometimes cooperates with this bacterial species, *S. aureus* eventually dies in the presence of *P. aeruginosa*. This happens when *S. aureus* alters metabolic gene expression, affecting key metabolic pathways and reducing the energy used to survive. In these cases, the genes involved in nucleotide metabolism (nrd operons) are downregulated, limiting DNA synthesis, DNA repair control, and affecting *S. aureus* cell concentration [137],[139]. The switch from aerobic respiration to lactic acid fermentation allows *P. aeruginosa* to consume the lactate produced by *S. aureus* as a carbon source [160]. Moreover, genes involved in glycolysis and the pentose phosphate pathways are downregulated in *S. aureus*, revealing that there is a competition for nutrients [137],[139], which *S. aureus* loses. In *P. aeruginosa*, nitrogen starvation leads to the expression of *ntrC*, a response regulator to nitrogen limitation, and to the upregulation of genes involved in nitrogen assimilation, including those encoding glutamate dehydrogenase and synthase. On the other end, in *S. aureus*, nitrogen starvation leads to a downregulation of the glutamate synthase genes. Therefore, the additional availability of carbon and nitrogen provided by *S. aureus* may be beneficial for *P. aeruginosa*, allowing it to gain an advantage over other bacteria during colonization and during long-term chronic infections [25],[157],[177].

Additionally, in the presence of *P. aeruginosa*, *S. aureus* releases the peptidoglycan component N-acetyl glucosamine (GlcNAc) upon cell lysis or cell wall turnover during growth, which in turn inactivates the *P. aeruginosa* caseinolytic protease ClpXP. This protease has a critical role during quorum sensing homeostasis, leading to an increased production of virulence factors by *P. aeruginosa*, such as pyocyanin and exotoxins, that can damage *S. aureus* [22],[162],[194]. Moreover, lysed *S. aureus* cells might be a source of iron for *P. aeruginosa* [195]. Furthermore, the non-motile phenotype of *S. aureus* may bring some disadvantages during infection and colonization. Samad *et al*. [196] observed that the *S. aureus* location changed together with the one of *P. aeruginosa* by moving upwards along with the swimming cells of *P. aeruginosa* , leading to a proximity that can be harmful to *S. aureus*.

*P. aeruginosa* can also indirectly prejudice *S. aureus* by manipulating the innate immunity of the host (e.g., by inducing the production of phospholipase sPLA2-IIA by bronchial epithelial cells), which leads to *S*. *aureus's* death [197]. This may be simply a response of the host to which *P. aeruginosa* resists; however, it suggests that *P. aeruginosa's* interactions with the host can disrupt bacterial communities more broadly than previously expected [135],[198].

#### 5.1.1 *P. aeruginosa* and *S. aureus* interactions lead to increased antimicrobial resistance

*S. aureus* and *P. aeruginosa* normally show high antimicrobial resistance toward multiple compounds [199]. According to the European Center for Disease Control, from 2020 to 2022, approximately 30% of the *P. aeruginosa* isolates were resistant to at least one of the antimicrobial groups under surveillance [200]. According to Mottola *et al*. [199], 36% of fifty-three staphylococci isolated from DFU cases were multidrug-resistant. When bacteria like *P. aeruginosa* and *S. aureus* are present together in biofilms, resistance to antibiotics and to the human immune system can be up to 1,000 times higher than the one showed by their planktonic counterparts [199],[201],[202]. In chronic infections, hypoxia and thick dehydrated mucus provide optimal conditions for biofilm formation and contribute to adaptive resistance [1],[203]. Moreover, the proximity between bacteria in biofilms contributes to horizontal gene transfer and further antimicrobial resistance.

It was found that, in the presence of *P. aeruginosa*, *S. aureus* gains resistance to tetracycline and ciprofloxacin [137]. According to Briaud *et al*. [137], *P. aeruginosa* induces the over-expression of the Nor family genes (*norA*, *norC*, and of *tet38*), which encode efflux pump transporters responsible for increased antibiotic resistance in *S. aureus*. The most over-expressed gene is *tet38*, implicated in resistance against tetracycline. In addition, this transporter interacts with the CD36 receptor on epithelial cells, increasing *S. aureus* internalization, helping it to hide in epithelial cells and escape the attack from either the immune system or from *P. aeruginosa* [204].

Recent studies also suggest that *S. aureus* can enhance *P. aeruginosa's* antibiotic resistance ability [13],[205]. Beaudoin *et al*. [13] have demonstrated that the interaction between *S. aureus* SpA and *P. aeruginosa* Psl increases tobramycin resistance in *P. aeruginosa*. In the presence of *S. aureus*, this bacterial species synthesizes truncated lipopolysaccharides that lack a O-specific antigen and contribute to increased resistance to β-lactam antibiotics, but not against ciprofloxacin or polymyxin [205]. These studies show that *P. aeruginosa* is able to acquire resistance mechanisms against antibiotics targeting the cell wall in polymicrobial biofilms, such as β-lactams, and in protein biosynthesis, such as tetracycline [13],[160].

Regarding *S. aureus* SCV, they exhibit an intrinsic tolerance to antibiotics, which is not necessarily associated with antimicrobial resistance genes [206]. In this process, the shift to fermentative metabolism results in decreased ATP production, reducing the active transport of antibiotics that inhibit protein synthesis, such as tetracycline, aminoglycosides, and macrolides [160],[166],[170],[176],[177]. When *S. aureus's* intracellular ATP is reduced, it slows down cell proliferation, decreasing *S. aureus's* susceptibility to cell wall-targeting antibiotics [176],[177]. The reduced membrane potential and higher persistence of SCV contribute to antibiotic tolerance [170],[187].

### 5.2. P. aeruginosa and B. cepacia complex

*B. cepacia* complex, or simply *B. cepacia*, is formed by at least 20 different species, including *B. cepacia* and *B. cenocepacia*, which are Gram-negative, catalase-producing, and lactose-non-fermenting bacteria [207]. *B. cepacia* seldom causes problems in healthy persons, as it is an opportunistic pathogen that is associated with pneumonia mainly in immunocompromised individuals with an underlying disease, like CF or the immunodeficiency chronic granulomatous disease [208].

Eventually, if *B. cepacia* complex bacteria gain access to lungs previously infected with *P. aeruginosa*, the close interactions between both bacterial species may influence virulence and result in a more rapid decline of human pulmonary function and eventually in death [78],[135]. Competition between these two bacteria is intense, as populations of both species have been found to be able to invade each other [51],[209],[210]. In a study on CF, most clinical isolates of *P. aeruginosa* (81%) and *B. cenocepacia* (57%) were found to be able to secrete bacteriocin-like toxins, such as pyocyanin, inhibiting each other's growth [211],[212].

Cooperative mechanisms between both species can also be detected, mainly benefiting *B. cepacia* [135]. When in coculture, *P. aeruginosa* upregulates putative *B. cepacia* virulence factors and increases its adhesion to the host epithelium [213]. Additionally, N-acyl homoserine lactones produced by *P. aeruginosa* can stimulate the production of lipases, proteases, and siderophores by *Burkholderia*, promoting its growth [210],[213]–[215]. Siderophores are very important elements in bacteria, since they are high-affinity, iron-chelating compounds for iron uptake, an element essential for growth, as several metabolically important proteins in living cells depend on iron [214],[215]. Finally, alginate production by *P. aeruginosa* is known to inhibit the host immune system and indirectly aid *B. cenocepacia* survival [216].

### 5.3. P. aeruginosa and SMG members

Members of the SMG, also known as the *Streptococcus anginosus* group which include *S. constellatus*, *S. intermedius*, and *S. anginosus*, are gram-positive facultative anaerobic, catalase-negative, and nonmotile cocci [217]. They are common members of the airway microbiota and the genitourinary and gastrointestinal tracts, but can be involved in purulent infections, often resulting in abscess formation, which distinguishes them from other pathogenic streptococci, such as *S. pyogenes* and *S. agalactiae* [218]. SMG has recently been associated with clinically relevant pulmonary exacerbations in CF patients [84],[219].

Interestingly, *in vitro* investigations showed that *P. aeruginosa* differentially expresses several genes related to virulence factors and drug efflux pumps when in co-culture with streptococci [80]. Furthermore, the expression of some of these genes could be mediated by the streptococci quorum-sensing molecule AI-2 (section 4.1), which was found in high levels in the sputum of some CF patients. This result suggests that communication between microbes of the commensal microbiota through quorum sensing may exacerbate CF disease caused by conventional pathogens like *P. aeruginosa* [11],[80].

### 5.4. P. aeruginosa and fungi: A. fumigatus and C. albicans

The fungus *A. fumigatus* is one of the most common *Aspergillus* species causing disease in immunocompromised individuals. It is the most common fungus found in CF airways [144], and the clinical presentation of associated diseases may include no apparent respiratory failure, bronchitis, and bronchiectasis [82],[220]. Infection tends to occur after *P. aeruginosa* colonization, resulting in synergistic co-infections that cause more severe clinical outcomes compared with those promoted by each microorganism alone [135],[146],[221].

When they coexist in the CF lungs, the interactions between *P. aeruginosa* and *A. fumigatus* are extensive and mainly concerned to the production of *P. aeruginosa* phenazines, which are beneficial to the fungus. It is known that high concentrations of phenazines, including pyocyanin, phenazine-1-carboxylate, phenazine-1-carboxamide, and 1-hydroxyphenazine, inhibit *A. fumigatus* growth by inducing the production of reactive oxygen and nitrogen species [222],[223]. However, pyocyanin, phenazine-1-carboxylate, and phenazine-1-carboxamide are usually present in subinhibitory concentrations *in vivo*, promoting fungal growth by acting as iron-reducing agents. They sequester ferric iron (Fe^3+^), reducing it into the more soluble ferrous iron ions (Fe^2+^), enhancing iron uptake in the host where iron is tightly bound to proteins such as hemoglobin and with extremely limited availability [223]. It was also shown that the *P. aeruginosa* phenazine 1-hydroxyphenazine causes iron starvation of *A. fumigatus* by chelating iron, which is partially compensated by the fungal mechanisms adapted to iron starvation, such as the production of siderophores [135],[223].

During chronic infections, damaged host cells release iron in the form of haem groups and hemoglobin. In these infections, phenazine-1-carboxylic acid present in infected tissues may shift the redox equilibrium between Fe^3+^ and Fe^2+^, thereby helping to make iron become more bioavailable [224]. There is some evidence that *P. aeruginosa* siderophores, pyoverdine and pyochelin, are replaced by a haem assimilation system used by *Pseudomonas* to sequester iron, generating an iron-rich environment [225].

*A. fumigatus* infections generally occur after *P. aeruginosa* colonization because a) *P. aeruginosa* previously generates an iron-rich environment in which *A. fumigatus* can prosper; and/or because b) “damaged lungs” allow for better and faster colonization by pathogens [135].

The fungus *C. albicans* is a common, commensal microbe that colonizes the oropharyngeal cavity, gastrointestinal and vaginal tracts, and skin of healthy individuals without causing disease. The transition from an element of the commensal microbiota to an agent of opportunistic infections depends on different factors. Opportunistic *C. albicans* can be found in superficial mucocutaneous disorders, in parenteral nutrition feeding tubes [77], in denture stomatitis [73] and dental caries [72], and in CF [226]. *C. albicans* is a dimorphic yeast, and can switch from a round yeast, generally associated with commensal form, and germinate to an elongated hyphal form, generally associated with opportunistic infections. This transition is central to its pathogenesis [11],[226].

In CF patients, *C. albicans* is frequently found in the lower airways due to the high amounts of fungus colonizing the nasopharynx, mainly due to repeated rounds of antimicrobial therapy [143]. In its pathogenic hyphae form, the elongated structure is able to puncture the epithelial and endothelial layers to gain access to deeper tissues, which is crucial for biofilm formation [227],[228].

When coexisting, the interactions between *P. aeruginosa* and *C. albicans* are essentially antagonistic, generally towards the fungus [11]. These interactions are complex, and their influence on the human host is not fully understood. Studies revealed that when in co-culture, *P. aeruginosa* is able to inhibit *C. albicans* germination by secreting 3-oxo-C_12_ homoserine lactone, decreasing *Candida* pathogenic potential [145]. However, the secretion of this molecule diminishes during chronic infections [229], and consequently prompts the development of the invasive filamentous form of the fungus [230]. Moreover, *P. aeruginosa* can attach to specific areas of the *C. albicans* hyphal surface, effectively eliminating the hyphal cell by inducing its lysis, though it is incapable of acting on the round yeast form of the fungus [11],[31].

Lower concentrations of *P. aeruginosa* phenazines in polymicrobial biofilms inhibit respiration and promote fermentation by *C. albicans*, increasing the production of products like ethanol, glycerol, and acetate. These metabolic effects impair the *C. albicans* biofilm formation and the fungal transition to the hyphal form [231]. In turn, ethanol leads to the enhanced development of *P. aeruginosa* biofilms and the formation of a more virulent mucoid phenotype [231],[232]. Furthermore, ethanol is an immunosuppressant, indirectly influencing the number and diversity of microbes within the polymicrobial biofilm [233],[234]. Ethanol has similar effects on other lung associated pathogens such as *S. aureus* [235] and *A. baumanii* [236], though the mechanisms behind these events have not yet been fully identified [135].

Nevertheless, interactions between *P. aeruginosa* and *C. albicans* are not unilaterally antagonistic. *C. albicans* produces the quorum-sensing alcohol farnesol, which inhibits the transition from yeast to hyphae, resulting in fewer hyphal forms. Moreover, the secretion of farnesol has been shown to restrain the swarming capacity of *P. aeruginosa*, which may hamper the preliminary deposition of *P. aeruginosa* onto the hyphae surface, resulting in lower lytic activity [11],[230]. According to Cugini *et al*. [237], farnesol leads to a substantial downregulation of the *P. aeruginosa pqsA* gene, which is a mediator of pyocyanin expression. Therefore, during the close coexistence of both microorganisms in different diseases, farnesol secretion may be protective for *C. albicans* by reducing *P. aeruginosa* pyocyanin levels [238]. Finally, *C. albicans* can reduce the expression of *P. aeruginosa* siderophores, namely of pyoverdine and pyochelin, limiting its growth and further virulence [239].

### 5.5. P. aeruginosa and viruses

In a previous study, rhinovirus, influenza A virus, and influenza B virus were found to be the most frequent viruses colonizing the upper respiratory tract of children affected by temporary exacerbated CF, while the most common bacteria were *P. aeruginosa* and *S. aureus* [240]. In an *in vitro* work that studied the influence of RSV on the adherence of *P. aeruginosa* to epithelial cell monolayers, results showed that the presence of RSV increased adherence and further colonization of the host by this bacterial species during CF [83]. Another study aiming to determine if RSV could facilitate the development of acute infections by *P. aeruginosa in vivo* showed that a co-infection with both microorganisms lead to a paramount, nearly two-thousand-fold increase in bacteria colony counts in lung homogenates when compared to results from *P. aeruginosa* monomicrobial infection [241]. Therefore, viruses infecting the upper respiratory tract result in an exacerbation of CF symptoms [83],[240],[241], probably by serving as a bridge between the bacteria and host surfaces through the induction of a T-cell chemoattractant, named monokine, induced by gamma interferon [241]. They also exert immunomodulatory effects that inhibit bacterial clearance processes in the CF lung [241],[242].

## 6. Conclusions

Bacteria, fungi, and viruses are often co-isolated together from complex polymicrobial biofilm communities *in vivo* and are responsible for clinical and pathological manifestations in humans. It is known that microbial communities within biofilms are spatially organized in either a mixed or segregated way and establish either physical or chemical interactions between them. These interactions can exhibit either cooperative or antagonistic effects, either benefiting or not benefiting the community, and may result in a synergistic outcome influencing bacterial clearance mechanisms. They can affect virulence directly or indirectly, modifying the clinical course of the disease, with biofilms being intimately associated with disease progression and worsening of patient's symptoms. *P. aeruginosa* is hard to eliminate as it can express several efflux systems and present the capacity to adapt to several environmental conditions, being the cause of many human diseases. In the presence of other microorganisms, it can establish interrelationships mostly beneficial to itself, forming strong biofilms that are even more resistant to the host immune system and to antimicrobial activity. Therefore, during the evaluation of a given disease, it is of utmost importance to determine if the infection is caused by polymicrobial biofilms and which microbes are involved, as their presence has significant implications for disease management. For example, the presence of antimicrobial resistant bacterial pathogens will influence the selection of antimicrobial therapy. As such, in future studies the challenge will be to identify potential targets for the inhibition of biofilm co-adhesion and development into large intraspecies and interspecies complex networks inside the human host.

## Use of AI tools declaration

The authors declare they have not used Artificial Intelligence (AI) tools in the creation of this article.
